# Summary of the Course on Demonstrative Surgery in the University of Pennsylvania, March 1st, 1851

**Published:** 1851-04

**Authors:** 


### CLINICAL REPORTS.

### *Summary of the course on Demonstrative Surgery in the University of Pennsylvania, March* 1*st*, 1851.

(Reported for the Examiner.)

At the close of our last paper, a brief reference was made to some of the cases shown at the end of the hour, and especially to a rare form of dislocation of the femur. As the demonstrative course terminates on the 1st of March, in order to facilitate the arrangements connected with the extension of the term of lectures to the 1st of April, we have only the cases of this day and the details of the dislocation to report.

#### *Large Tumor in the Ham.*

The first patient brought forward by Dr. Smith on the 1st of March, was an example of some of the difficulties attendant on the diagnosis of swellings found in the Popliteal Region. The patient, aged about 30, was a day laborer, and whilst engaged, Sept. 1850, in carrying corn to the third story of a warehouse, felt something give way behind his left knee joint. This was soon followed by pain and swelling to such a degree, as to interfere with his ordinary labor. A few days afterwards, when Dr. Smith saw him, the tumor was about the size of a small egg; situated on the inner side of the ham; not moveable; slightly elastic; not painful on pressure, but with an obscure sense of pulsation and fluctuation. From the difficulty of deciding as to its character, a palliative treatment was adopted, with but little relief and without arresting the increase of the tumor. On his re-appearance to day, the tumor was found to be larger than an orange; quite tense; evidently beneath the fascia; could be partly diminished by pressure, but was now free from pulsation. Another small tumor, similar in appearance to this, at its commencement, was now also found in the right ham ; and the right elbow beneath the triceps tendon presented something of the same kind, with stiffness of the joint, perfect extension being impossible. Being satisfied, therefore, that no aneurism existed in the ham, but that the disease was a bursal tumor, opening into the knee joint, the Dr. determined to evacuate its contents by a valvular puncture, and be guided in the after treatment by circumstances. With this view he introduced a small trocar and canula beneath the skin; carried it to a short distance from the point of entrance; tapped the tumor on the opposite side to that where the skin was opened, and drew off over four ounces of clear straw-colored synovial fluid. The instrument being then carefully withdrawn, so as to exclude the air, the opening was closed. In about ten days the cyst again filled, and the patient is yet under treatment.

*Hard and Soft Cataract.*

The next patient shown, was an elderly man from Montgomery country, who was suffering from double cataract, one of them being of fifteen years standing, and both apparently soft in their consistence. The pupils being well dilated by belladonna, Dr. Smith determined to operate on both eyes, and with the left hand introduced the needle into the right eye, lacerated the capsule and evacuated a softened lens, enabling the patient to see immediately. Then changing the needle, he introduced it with the right hand into the left eye, and finding the cataract firm and hard, depressed the lens out of the axis of vision, so as to enable the patient to see with both eyes before leaving the lecture room. Subsequntly the symptoms were of a trifling character, and the inflammation quite moderate. The patient's pupils are now entirely clear.

*Case of Vesical Calculus, removed by Lithotomy.*

On the removal of this patient, Dr. Smith next presented a man laboring under stone in the bladder, of about two years duration. This patient, aged forty-three, had lived in Philadelphia for the last seventeen years, and previously in Ireland; knew no cause for his disease except intemperance, and this he ascribed, during the last eighteen months, to his sufferings since the formation of the calculus. At one time he had drank over one pint and a half of whiskey per diem, but for the last six months had taken much less, and lately abstained almost entirely. He had all the symptoms of stone well marked, his constitution was giving way under suffering and bad habits, and there was also considerable ossification of his arteries. Believing from the apparent character of the stone as shown in sounding, from the general symptoms, and from his domestic circumstances, that lithotripsy was not adapted to the case, a consultation had ad vised him to submit to lithotomy. Sounding satisfied every one who was near, that the stone was a hard one, as with silence in the room, the click of the sound could be heard some distance. It was also deemed of considerable size, and Earle's forceps were kept in readiness for service if it should be necessary to crush the calculus, previously to its extraction. The patient having been, therefore, as well prepared by previous treatment as was possible, was placed upon the table in the ordinary position for lithotomy, and cut by Dr. Smith, (assisted by Drs. Horner, Wallace, Neill and Page,) by the usual lateral operation and the gorget. No anæsthetic was resorted to, as it was deemed unadvisable to attempt etherisation in the case of one so habituated to liquor, and with such disease of his circulatory organs. On opening the bladder, the stone was found to be attached to the left side of the viscus, and very rough and large, rendering it a difficult matter to place it fairly in the grasp of the forceps. Considerable care therefore was necessary in freeing it from its attachments and extracting it, but by cautious manipulation and further dilation with the bistoury, this was successfully accomplished. The operation, including the period consumed in the efforts at extraction, (which constituted the great difficulty,) occupied 17 minutes. On examination, the stone proved to be a rough and large one, measuring two and one-eighth inches in its long diameter, and one inch and five-eighths in its transverse measurement. On chemical analysis by Dr. Rogers, the Professor of Chemistry, it was found to be phosphate of ammonia and magnesia, together with a small amount of phosphate of lime, with a nucleus the size of an English walnut, resembling a mulberry calculus. Its weight was ℥ij. gr. xvii. besides some portions crushed in the efforts at extraction and lost. From the thickness of the perineum and the ossified state of the blood vessels, the hemorrhage was free from branches of the hemorrhoidal and transversa perinei, each of which required a ligature. A catheter being then left in the wound, the man was placed in bed as usual and rallied well from the operation. Twelve hours afterwards, the urine ran free and clear through the catheter, and he has not since had a bad symptom. Much of his comfort and favorable condition has been due to the devoted attentions of the members of the class,* who nursed him most sedulously for some time after the operation. The urine has now passed through the urethra, the perineal wound is healing, and he is now (March 25th,) nearly well.

*Amputation of the Breast.*

Immediately after this patient was carried to his bed, Dr. Smith introduced a lady who had been laboring for several months under scirrhus of the mamma of the right side, the result apparently of inflammation and thickening consequent upon suckling. During lactation, four years since, she had suffered from a milk abscess, and from the irritation consequent upon this, scirrhus had developed itself. The tumor was now about the size of an apricot, accompanied by a tendency to retraction of the nipple, and the other well marked signs of Cancer. After moderate etherisation the Dr. removed the breast in the usual manner, extending the dissection slightly into the axilla, in order to free it from all possible points of diseased structure. The wound was then dressed in the ordinary manner, and has healed rapidly.

*Messers Vogdes, Hempstead, Little, Jackson, Wilson, Horner, Ware, Kieffer, &c.

These operations having occupied the hour, the demonstrations were closed by a few valedictory remarks by Dr. Horner.

*Dislocation of the Femur directly backwards.*

In referring to the rare character of the dislocation in the last report, want of space prevented our presenting its details. They are as follows: A man aged about 35, whilst assisting to push a line of coal cars, was jammed with a companion between two of them. As well as he can recollect, he was struck on the buttock and jammed against the front car. His companion was killed, the patient owing his life to the death of his fellow-laborer. The terrible contusion, swelling, &c., which continued for three weeks after the accident, prevented anything like an accurate diagnosis, though a fracture of the neck of the femur was suspected. At the end of five weeks, as he suffered much pain in the hip and down the limb, and was unable to bear his weight on the leg, a further and more minute examination by Dr. Horner, detected a dislocation of the head of the femur upon the ischium, between the spine and the tuberosity. The signs were as follows:—1st, increased width and flatness of this buttock. 2d, A hard round tumor (head of bone) beneath the gluteus magnus muscle, or rather about the junction of the gluteus medius and magnus. 3d, Very slight shortening, the heel not touching the ground when the pelvis was held firmly. 4th, Foot apparently natural when in bed. 5th, When standing erect, toes can be turned to metatarso-phalangeal articulation of opposite foot and no further. 6th, Toes can be turned out about thirty degrees, and no more. 7th, Abduction of femur imperfect. 8th, Adduction nearly perfect, as also flexion and extension.

*Summary of the symptoms.*—Perception of head of femur and its motion, on motion of limb. Very slight change in the length of limb. Very slight variation of line of toes, but impossibility of fully inverting and especially everting the foot. Two days after use of pullies as before detailed, eversion of foot possible to 90 degrees; inversion perfect; improved rotundity of buttock; re-appearance of the hollow of the buttock, just above the trochanter major; abduction of thigh easy, and patient fully sensible of a change in the state of the part as well as of more freedom in his motions.

